# Design and computational analysis of single-cell RNA-sequencing experiments

**DOI:** 10.1186/s13059-016-0927-y

**Published:** 2016-04-07

**Authors:** Rhonda Bacher, Christina Kendziorski

**Affiliations:** Department of Statistics, University of Wisconsin, Madison, WI 53706 USA; Department of Biostatistics and Medical Informatics, University of Wisconsin, Madison, WI 53726 USA

## Abstract

Single-cell RNA-sequencing (scRNA-seq) has emerged as a revolutionary tool that allows us to address scientific questions that eluded examination just a few years ago. With the advantages of scRNA-seq come computational challenges that are just beginning to be addressed. In this article, we highlight the computational methods available for the design and analysis of scRNA-seq experiments, their advantages and disadvantages in various settings, the open questions for which novel methods are needed, and expected future developments in this exciting area.

## Background

The ability to derive genome-wide mRNA expression data from a population of cells has proven useful in thousands of studies over the past two decades. In spite of their utility, traditional expression experiments are limited to providing measurements that are averaged over thousands of cells, which can mask or even misrepresent signals of interest. Fortunately, recent technological advances now allow us to obtain transcriptome-wide data from individual cells. This development is not simply one more step toward better expression profiling, but rather a major advance that will enable fundamental insights into biology.

**Fig. 1:**
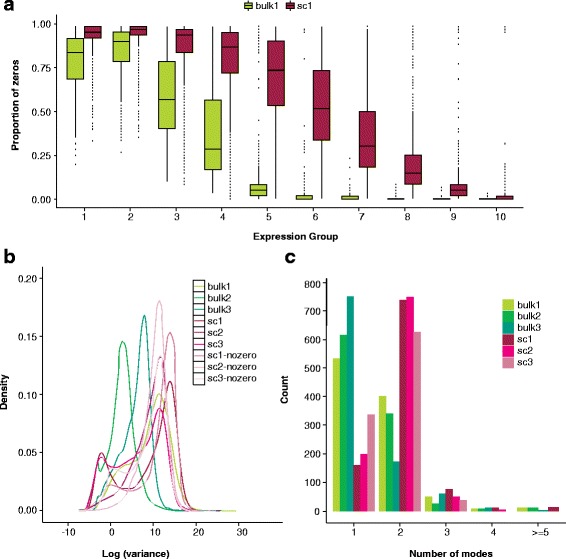
Prominent features in single-cell RNA-seq data relative to bulk RNA-seq include an abundance of zeros, increased variability, and multi-modal expression distributions. **a** Boxplots of the gene-specific proportion of zeros in a bulk (*bulk1*) and single-cell (*sc1*) dataset stratified by percentile of median gene expression. Sequencing depth ranges from 420,000 to 16.6 million in bulk1 and 385,000 to 16.4 million in sc1 (samples were chosen to have comparable depths; see the “Data” section). **b** Densities of gene-specific log variance for all genes in three bulk and three single-cell RNA-seq datasets. Densities are also shown for the single-cell datasets for log variances calculated following the removal of zeros, emphasizing that the increased variability observed relative to bulk is not entirely due to the presence of zeros. **c** For each dataset shown in **b**, 1000 genes were selected at random from the list of genes for which at least 75 % of cells showed non-zero expression. For each gene, zeros were removed and *Mclust* [92] was applied to log expression to estimate the number of modes. Because zeros were removed prior to *Mclust*, a mode at zero will not contribute to the total number of modes shown

While the data obtained from single-cell RNA-sequencing (scRNA-seq) are often structurally identical to those from a bulk expression experiment (some *K* million mRNA transcripts are sequenced from *n* samples or cells), the relative paucity of starting material and increased resolution give rise to distinct features in scRNA-seq data, including an abundance of zeros (both biological and technical), increased variability, and complex expression distributions (Fig. 1). These features, in turn, pose both opportunities and challenges for which novel statistical and computational methods are required.

In this review, we discuss such statistical and computational methods in detail. We begin with an overview of practices for robust experimental design, quality control, and expression estimation, where the principles and methods used in bulk experiments, perhaps slightly modified, apply directly. We then discuss methods for normalization, noting that features that are unique to scRNA-seq pose challenges for existing approaches. Methods developed for downstream analysis are also considered with specific focus on methods for clustering and sub-population identification, assessing differences in gene expression across conditions, pseudotime ordering, and network reconstruction (a summary is provided in Table 1). We conclude with a discussion of the open questions facing computational scientists, as well as those that will soon arise as datasets quickly become ever larger and more complex.

**Table 1:** Statistical methods for single-cell RNA-seq experiments

Name	Description	Requirements/deliverables
Normalization		
GRM [57]	Fits polynomial gamma regression model to FPKM data from spike-ins; estimated parameters are used to convert FPKM of endogenous genes to an absolute scale within each cell	Performs within cell normalization and may be used with FPKM, RPKM, or TPM
SAMstrt [56]	The resampling-based bulk normalization method in SAMseq is applied to spike-ins	Assumes that an equal number of spike-in control RNA molecules have been added to all samples
Identifying highly variable genes		
Brennecke et al. [48]	A gamma generalized linear model fit to the mean-variance relationship quantified by the square of the coefficient of variation (CV^2^) of the spike-ins estimates technical noise parameters. These parameters are then used to estimate technical variability for endogenous genes and to test whether each gene exceeds a variability threshold	Spike-ins and endogenous genes are normalized separately using the median normalization method. Gene specific *P* values are provided to identify highly variable genes
Kim et al. [63]	Uses spike-ins to estimate parameters related to technical variance, allowing for differences in variability across cells. Estimates gene-specific biological variability by subtracting technical variability from total variance	Normalization factors are estimated using the median normalization method. A simulation based framework to test for highly variable genes is provided
BASiCS [54]	Jointly models spike-ins and endogenous genes as two Poisson-Gamma hierarchicalmodels with shared parameters	Estimates normalization parameters jointly across all genes. Gene-specific posterior probabilities are provided to identify both lowly and highly variable genes
Noise reduction		
scLVM [47]	Uses a Gaussian Process Latent variable model to estimate the covariance matrix associated with latent factors. Residuals from a linear mixed model with the covariance term represent de-noised expression estimates	Requires genes associated with the latent factor to be identified a priori. Normalization factors are estimated using the median normalization method
OEFinder [12]	Uses orthogonal polynomial regression to identify genes whose expression is associated with position on the C1 Fluidigm integrated fluidic circuit (IFC)	Gene-specific *P* values are provided to identify genes affected by the artifact
Sub-population identification		
ZIFA [70]	Models dropout rate as a function of expression in a factor analysis (linear dimension reduction) framework	Requires normalized, log-transformed estimates of gene expression (zeros are not transformed)
Destiny [81, 82]	Extends diffusion maps (a non-linear dimension reduction approach) to handle zeros and sampling density heterogeneities inherent in single cell data	Requires variance-stabilized gene expression estimates; works best with a large number of cells
SNN-Cliq [71]	Clusters cells by identifying and merging sub-graphs (quasi-cliques) in a shared nearest neighbor (SNN) graph; the number of clusters is chosen automatically	Requires a reduced set of genes. Xu and Su [71] recommend using genes with average RPKM >20 and using a log transformation to reduce the effect of outliers. Relies on a valid choice of graph parameters
RaceID [59]	Uses k-means applied to a similarity matrix of Pearson’s correlation coefficients for all pairs of cells; the number of clusters is chosen using the gap statistic. Outlier cells are those that cannot be explained by a background model that accounts for technical and biological noise. In a second step, rare subpopulations can be identified and outlier cells may be merged to an outlier cluster; new cluster centers are then computed and each cell is assigned to the most highly correlated cluster center	Requires a reduced set of genes. Grün et al. [59] consider genes with a minimum of five transcripts in at least one cell
SCUBA [73]	Uses k-means to cluster data along a binary tree detailing bifurcation events for time-course data. Models expression regulation along the tree using bifurcation theory	Requires a reduced set of genes. Marco et al. [73] recommend using the 1000 most variable genes that are expressed in at least 30 % of cells
BackSPIN [60]	Iteratively splits a two-way sorted (by both genes and cells) expression matrix into two clusters containing independent cells and genes, for a maximum number of splits. The algorithm has a stopping condition to avoid splitting data that are very homogeneous	Requires a reduced set of genes and the maximum number of splits allowed. Zeisel et al. [60] recommend selecting the top 5000 genes that have the largest residuals after fitting a simple noise model
PCA/t-SNE [69]	Linear/non-linear dimension reduction approach used for unsupervised clustering of cells	Input is typically a correlation or similarity matrix
PAGODA [68]	Allows for both detection and interpretation of the transcriptional heterogeneity within a cell population. A weighted principal component analysis (PCA) is conducted for each gene set; those sets for which the variance explained by the first principal component significantly exceeds genome-wide background expectation are identified. To provide a non-redundant view of heterogeneity structure, principal components from different gene sets showing high similarity are combined to form a single component of heterogeneity	Requires un-normalized gene expression counts (performs internal correction as in SCDE). Uses gene ontology (GO) annotated or user-defined gene sets
Differential detection		
MAST [76]	A logistic regression model is used to test differential expression rate between groups while a Gaussian generalized linear model (GLM) describes expression conditionally on non-zero expression estimates. Models are corrected for cellular detection rate	Requires normalized gene expression estimates and provides gene-specific *P* values from summing likelihood ratio or Wald tests from the two components
SCDE [77]	Models gene-specific expression as a two-component mixture: a Poisson component describes zero and a Negative Binomial describes non-zero measurements	Requires un-normalized gene expression counts (performs internal correction) and provides gene-specific posterior probabilities of differential expression (DE) between two biological conditions. Tests for DE are performed on non-zeros
scDD [78]	Models expressed counts as a Dirichlet process mixture (DPM) of normals to test for differentially distributed (DD) genes associated with multi-modality in the expressed component. Samples from the posterior further characterize the gene-specific distributional difference between two biological conditions to identify genes that are differentially expressed (DE), differ in the proportion of cells within modes (DP), differ in the number of modes (DM), or are both DE and DM (DB)	Requires normalized, log-transformed gene expression estimates and provides gene-specific *P* values (or a false discovery rate (FDR)-controlled list) of DD genes between two biological conditions. Each DD gene is then classified into a specific type of distributional difference
Pseudotemporal ordering		
Monocle [36]	Reduces data using independent component analysis (ICA) and constructs a minimum spanning tree (MST) to order cells in pseudotime	Requires normalized, log-transformed gene expression estimates and a reduced set of genes. Trapnell et al. [39] recommend identifying genes that are differentially expressed between time points or, if data at multiple time points are not available, choosing genes above a mean and variance threshold
Waterfall [80]	Unsupervised clustering is used to identify clusters of cells for which a putative ordering is determined on the basis of their relative location in a PCA plot. K-means clustering of single-cell transcriptomes on the PCA plot and an MST that connects cluster centers determines pseudotime	Requires normalized estimates of gene expression with outliers removed
Sincell [83]	A flexible R workflow for building cell hierarchies with multiple options for dimension reduction, clustering, and graph building. Allows the user to assess the similarity of graphs and performs resampling or random cell substitution with simulated replicates to assess the robustness of estimated hierarchies	Requires normalized, log-transformed gene expression estimates and a reduced set of genes. Juliá et al. [83] recommend identifying highly variable genes
Oscope [11]	Uses a paired-sine model and K-medoids clustering to identify groups of oscillatory genes. For each oscillatory group, an extended nearest insertion algorithm is used to construct the cyclic order of cells, defined as the order that specifies each cell’s position within one cycle of the oscillation of that group	Identifies groups of oscillatory genes, when present. Requires normalized gene expression and use of only high mean, high variance genes is recommended
Wanderlust [93]	Cells are represented as nodes in an ensemble of k-nearest neighbor graphs. For each graph, a user-defined starting cell is used to calculate an orientation trajectory by iteratively computing the shortest-path distance between cells. The final trajectory is an average over all graphs	Developed using single-cell mass cytometry data, which typically describe few genes (<50) and tens of thousands of cells

## Experimental design

Generally speaking, a well-designed experiment is one that is sufficiently powered and one in which technical artifacts and biological features that may systematically affect measurements are randomized, balanced, or controlled in some other way in order to minimize opportunities for multiple explanations for the effect(s) under study. Guidelines toward achieving this end have been reviewed for bulk RNA-seq studies [1, 2] and many of the same guidelines also hold for scRNA-seq. In short, to minimize potential artifacts and confounding, it is important to randomize or balance over as many factors as possible, ideally so that putatively interesting features that are observed in the data are not due to artifacts imposed during sample preparation and/or data collection. Although the specific factors that are important in any design are highly experiment-dependent, if multiple populations are being assessed, a primary candidate for randomization is the order with which cells from distinct populations are processed and libraries are constructed. If multiplexing is done, the assignment of barcoded samples should be randomized or balanced across multiple lanes to minimize potential lane effects.

While randomization should be carried out to the extent possible, it is important to point out that, in many cases, conducting a fully randomized experiment is not realistic. Limited samples, a fixed number of single-cell isolation platforms and sequencers, time constraints, and budgets often prohibit the theoretically ideal experiment from being realized in practice. In most cases, samples must be processed in multiple batches, with randomization occurring within batch. This is exemplified nicely in an experiment concerning embryonic development that profiles single-cell expression in oocytes and zygotes as well as in 2-cell, 4-cell, and 8-cell embryos [3]. In this study, within each batch, each developmental stage was represented and stages were randomized in an effort to ensure that batch and stage were not confounded. Further discussion of possible batch effects and a specific experimental design that reduces the confounding of batch effect with biological signal are given in Hicks et al. [4]. Specifically, they propose a design in which cells from the biological conditions under study are represented together in multiple batches, which are then randomized across sequencing runs, flow cells, and lanes as in bulk-RNA-Seq. With this design, one can model and adjust for batch effects that result from systematic experimental bias.

Experimental design considerations will also be affected by the various protocols and platforms available for scRNA-seq. Platforms for isolating single cells vary substantially with respect to capacity, cost, and time. Both Saliba et al. [5] and Kolodziejczyk et al. [6] review single-cell isolation practices in detail, with the latter including details on the more recent droplet methods. Additional consideration must be given to the protocols used for extracting RNA from each isolated cell and for its conversion to cDNA; common methods vary with respect to transcript coverage and strand specificity. Details are provided in reviews by Grün and van Oudenaarden [7], Saliba et al. [5] and Kolodziejczyk et al. [6]. Another issue concerns whether or not to include synthetic spike-ins (external transcripts added in known concentrations) or unique molecular identifiers (UMIs; short random sequences attached to individual cDNA molecules). While both have a number of theoretical advantages for normalization and expression estimation, practical challenges have prevented their routine use in scRNA-seq studies to date. In addition to the challenges detailed by Stegle et al. [8], spike-ins are typically added in an scRNA-seq experiment at very high relative concentrations and, consequently, they take up a relatively large proportion of reads, an important consideration during deliberations regarding experimental design. In addition, recent droplet technologies are not yet able to accommodate spike-ins. UMIs offer a great advantage in reducing noise resulting from amplification bias [9, 10], but protocols that implement UMIs sequence only the 5′ or 3′ end of each transcript, making them unsuitable for studies of isoforms or allele-specific expression.

Whatever the design, it is always beneficial (and requires almost no additional resources) to record and retain information on as many factors as possible to facilitate downstream diagnostics. Just as it is standard to check residuals following a linear regression, it should be standard in sequencing experiments to check that effects of interest are not confounded by variations in technician, sample processing date/time, reaction temperature, position on cell capture device, lane, batch, proportion of detected genes, and so on. Through such an analysis, Leng et al. [11] identified an artifact related to position on Fluidigm’s IFC array; once identified, the effects of such artifacts can be removed [12]. It is also important to note that the sources of variation in an scRNA-seq experiment are not yet completely understood, and there likely are systematic effects that will be important in scRNA-seq that have yet to be discovered. Novel methods to identify such factors are needed and are beginning to be developed [4].

While many of the design principles established for bulk RNA-seq hold in the single-cell setting, specific guidelines to define what is meant by 'sufficiently powered' in an scRNA-seq experiment are less clear. As with bulk-RNA-seq, guidelines will generally depend on the questions of interest.

While most studies do not address the question of determining the minimum number of cells required for a given task, identifying the sequencing depth at which the majority of human transcripts expressed in a cell, or population of cells, are detected is a question that has received considerable attention. The consensus is that, beyond one million reads, there is very little change (<5 %) in the number of reliably expressed genes detected in a cell [13]. In fact, the majority of genes seem to be detected at 500,000 reads; and over half are typically detected at 250,000 reads [13, 14]. Furthermore, Shalek et al. [15] demonstrated that one million reads is sufficient to estimate the fraction of detectably expressing cells within a population and also to estimate the mean and variance of a gene’s expression among detectably expressing cells.

More reads will be required for more refined tasks, such as fully characterizing transcript structure, estimating the expression of rare isoforms, or distinguishing cells on the basis of subtle differences. Fewer reads but larger cell numbers may be preferred when mapping out a large population, searching for rare but distinct cell types, or pooling cells in silico to obtain average gene-expression clusters. Guidelines have yet to be reported for these considerations, as well as for most analysis tasks such as sub-population identification and the identification of transcripts showing differential distributions across conditions. As with any power calculation, precise guidelines will depend not only on the task at hand but also on the signal-to-noise ratio inherent to a given system. Pollen et al. [14] have shown, for example, that 50,000 reads is sufficient for cell-type classification in a sample of 301 cells containing diverse cell types. Conversely, in a seemingly homogenous cell population, deeper sequencing may be required to detect heterogeneity that is due to rare subpopulations. Evaluating the trade-off that exists between sequencing depth and number of cells will also depend on budget and, albeit to a much lesser extent, on platform, protocol, base-pair length, and genome size.

## Quality control and expression estimation

Once reads from a well-designed experiment are obtained, quality control should be performed on the raw reads, on the aligned reads, and across the collection of cells in an effort to identify low-quality cells that should be removed prior to expression estimation. Low-quality refers to those cells that are broken or dead or to capture sites that are empty or contain multiple cells; a detailed discussion is provided in Ilicic et al. [16]. Microscopic inspection of capture sites is often used to identify and remove empty or multiple captures prior to sequencing [11, 17, 18], but such a visual inspection is not possible with all platforms, is not feasible in very large-scale experiments, and is not helpful in identifying subtle features associated with low quality [16]. Beyond visual inspection, many of the hallmarks of low quality are qualitatively the same as in bulk RNA-seq; consequently, existing tools are proving useful in the single-cell setting [8]. FASTQC [19], Kraken [20], and RNA-SeQC [21] are all popular tools for assessing the quality of raw and mapped reads within an individual sample. Each calculates read quality using summaries of per-base quality defined using the probability of an incorrect base call [22]. Cells with unusually high numbers of low-quality reads are flagged for removal. Graphical interfaces allow a user to assess quickly whether there is structure in the low-quality scores: an abundance of low-quality scores in the first few positions of many reads may indicate a transient problem with the run, whereas a decrease in quality in the last positions indicates a general degradation. Trimming may prove useful in the latter but is not suggested for the former. The FASTQC website discusses these and other issues in detail [19]. For samples with sufficiently high-quality reads, as evidenced by relatively few base-call errors, additional features should be assessed. For most genomes, in a complex library free of nucleotide composition, GC content, and/or amplification bias, the proportion of nucleotides should be approximately equal across read positions (at least after an initial bias that may be present due to certain priming protocols), GC content should be approximately normally distributed across reads with a mean and variance similar to that in the reference transcriptome and very few reads should be duplicated.

Additional criteria should be assessed once reads are mapped to a reference transcriptome. The most common metrics are total number or reads, number of transcripts sequenced or detected, the proportion of uniquely mapping reads, and the proportion of reads mapping to annotated exonic regions, where low numbers are indicative of sample degradation and/or bias. The proportion of reads mapping to the mitochondrial genome may also be useful in identifying low-quality cells because in a broken cell cytoplasmic RNA will be lost, while RNAs that are enclosed in the mitochondria will be retained [16]. If spike-ins are used, the ratio of reads mapping to synthetic and endogenous transcripts can be informative [23]. Specifically, a high ratio may indicate that a cell was broken during the capture process [16]. Patterns associated with coverage are also important [21, 24] and can be evaluated, for example, by considering the evenness of coverage as represented by the mean coefficient of variation across transcripts, 5′/3′ coverage as assessed by calculating the average coverage at each percentile of length from annotated 5′ and 3′ ends of known transcripts, and gaps in coverage. Levin et al. [24] discuss these metrics in detail and Li et al. [25] provide examples; RNA-SeQC provides a software package to facilitate straightforward calculation and visualization [21]. It is important to note that expected coverage patterns will depend on protocol and should be evaluated accordingly [7].

Given all the metrics potentially relevant in assessing a cell’s quality, it can be difficult to decide which samples to include. Specifically, what proportion of low-quality reads is considered unusually high? How many reads should be unique in a sufficiently complex library? FASTQC provides suggested thresholds that may be used for these and many of the other metrics discussed above. Although useful, thresholds will depend on many factors, including specific features of the transcriptome under study, read length, library preparation protocols, and the experimental design. For some measures, thresholds from bulk do not apply; mapping rates, for example, are typically lower in scRNA-seq. For these reasons, it can be helpful to compare metrics across many samples. QoRTs [26] and Qualimap2 [27] allow a user to assess the quality of individual cells in the ways just described, but also introduce metrics to assess quality across a collection of samples. This allows a user to identify outlier cells with respect to any of the metrics just discussed. It also allows for the identification of batch or other systematic artifacts that are not visible when considering individual samples in isolation. Caution must be exercised when discarding individual cells, or groups of cells, at this stage as a cell’s distinct features may be due to interesting biological processes and not technical artifacts. Keeping in mind that QoRTs and Qualimap2 were developed for bulk RNA-seq, in which outlier samples are more likely to be due to artifacts, extra caution should be exercised before discarding a cell that passes quality control individually but not in the group setting. Ilicic et al. [16] recently developed a supervised classification approach for identifying low-quality cells in the single-cell setting. Like QoRTs and Qualimap2, it considers a collection of cells; it also accommodates not only technical but also biological measures of cell quality and, because of its comprehensiveness, is likely to become one of the state-of-the art methods in this area.

With quality cells in hand, expression may be represented as counts from non-UMI data using HTSeq [28] or as expected counts using RSEM [29] or WemIQ [30]. If UMI-tagged data are available, counts can be obtained using approaches such as those detailed by Islam et al. [10] or Hashimshony et al. [31]. Measures of relative expression within a cell are also often used; these include transcripts per million mapped reads (TPM) or reads/fragments per kilobase per million mapped reads (RPKM or FPKM, respectively). As detailed below, these measures are not appropriate for comparing expression across cells in most cases because they assume that RNA content is constant across cells and that genes are equivalently expressed. For most downstream analyses, normalization among cells is needed.

## Normalization

Normalization commonly refers to adjusting for differences in expression levels that result from technical artifacts, so that expression may be compared within or between samples. It is widely recognized that many systematic sources of variation affect scRNA-seq read counts and *should* be adjusted for, including capture inefficiency, amplification biases, GC content, differences in total RNA content, sequencing depth, etc. In practice, however, it is difficult to estimate many of these variance sources and so most often scRNA-seq normalization amounts to adjusting for differences in sequencing depth. When well-behaved and representative synthetic spike-ins and/or UMIs are available, further refinement is possible. We first discuss methods for normalization that do not involve spike-ins or UMIs.

### Normalization without spike-ins or UMIs

A number of scRNA-seq studies normalize for sequencing depth within a cell by calculating TPM [14, 15, 23, 32, 33] or RPKM/FPKM [34–37]. Although useful, within-cell normalization methods are not appropriate for many downstream analyses because they do not accommodate changes in RNA content and they can be misleading when genes are differentially expressed [38]. A number of studies have demonstrated, albeit in the bulk RNA-seq setting, that between-sample normalization (adjusting for sequencing depth and/or other factors to make samples comparable across a collection) is essential for principal components analysis (PCA), clustering, and the identification of differentially expressed (DE) genes [39–41]. A striking example is provided by Bullard et al. [40], who show that the normalization procedure has a bigger effect on the list of DE genes than do the specific methods used for DE testing. Although these results were derived for bulk RNA-seq, it is clear that appropriate between-cell normalization will be just as important for single-cell analyses. Unless otherwise noted, we will hereinafter use normalization to mean between-cell normalization.

Given the importance of normalization, it is not surprising that many normalization methods are available for bulk RNA-seq experiments [40–46], and these methods have been used in the majority of reported scRNA-seq experiments to date. Specifically, many scRNA-seq studies use median normalization [47–51] or a similar method [52, 53]. Although the details differ slightly among approaches, each attempts to identify genes that are relatively stable across cells (not DE), then uses those genes to calculate global scale factors (one for each cell, common across genes in the cell) to adjust each gene’s read counts in each cell for sequencing depth or other sources of systematic variation. Scale factors are defined such that adjusted expression of the putative stable genes is relatively constant across cells. In other words, these methods assume that systematic variation among the stable genes is due to technical sources. Consequently, when that is not the case (for example, when there are global systematic shifts in expression resulting from changes in RNA content), these approaches can produce erroneous results [8]. In addition, most methods derived from bulk RNA-seq discard genes having any zero counts; and given the abundance of zeros in single-cell data, doing so can have major effects on normalized counts, with estimates of global scale factors becoming unstable [54]. Finally, global scale factor approaches assume that the relationship between read counts and sequencing depth is common across genes, which may not be the case in the single-cell setting.

### Normalization with spike-ins and/or UMIs

As mentioned above, global scale factors assume that RNA content is constant, which is often not the case in single-cell analyses as RNA content will vary with cell-cycle phase, cell size, and the transcriptional dynamics of select genes [55, 56]. Spike-ins, synthetic transcripts spiked into each cell’s library at known concentrations, can be used to estimate relative differences in RNA content and thereby improve normalization. The idea is that differences between the observed and expected expression of spike-ins can be attributed to technical artifacts. By calculating a cell-specific factor that adjusts for the differences, and by applying that factor to endogenous genes, normalized expression estimates can be obtained. Some scRNA-seq studies use spike-ins to improve estimates of global scaling factors [47] and statistical methods have been proposed for this purpose [54, 56, 57]. In spite of the promise, there are many challenges in getting spike-ins to work well, which can result in inconsistent detection [9, 17] (details are provided in Stegle et al. [8]). As a result, the use of spike-ins in scRNA-seq is not routine. UMIs are another control that holds much promise. In short, random sequences are attached to individual molecules prior to PCR, making each molecule unique and allowing for an absolute molecular count [10, 58]. UMIs have been successful in greatly reducing amplification noise in scRNA-seq data [9, 10, 59–62], but they cannot be used in studies of isoforms or allele-specific expression [8]. As with spike-ins, their use in scRNA-seq is not yet routine. In summary, due to a lack of methods that can accommodate features inherent in single-cell data and the challenges in routinely generating high-quality, representative spike-ins, improved methods for normalization of scRNA-seq data are required.

## Estimating and adjusting for nuisance variation

Several strategies have been proposed to reduce noise from both technical and biological sources in scRNA-seq experiments [9, 49]. In spite of considerable progress, challenges remain, and scRNA-seq protocols continue to have substantially increased levels of nuisance variation relative to bulk RNA-seq. Capture efficiency (percentage of mRNA molecules in the cell lysate that are captured and amplified), amplification bias (non-uniform amplification of transcripts), and sequencing efficiency (rate at which cDNAs in a library are sequenced) are major contributors to technical variation. These sources affect counts in both a gene- and a cell-specific manner and are observed to have the greatest effect on lowly expressed genes [48, 63, 64]. Considerable variation also results from differences among cells in cell-cycle stage or cell size, variation that is not typically observed in (unsynchronized) bulk RNA-seq experiments in which expression is profiled on average over thousands of cells. These biological sources of variation are not of interest in most experiments and hence contribute to nuisance variation, although we note that in some experiments (for example, investigations of cell-cycle genes), this variation will be of direct interest. Given the substantial variability present in scRNA-seq measurements, separating nuisance from meaningful biological variation is crucial for accurately characterizing sub-populations, identifying highly heterogeneous genes, and comparing expression levels among groups of cells; a number of statistical approaches have been developed toward this end.

One group of methods aims to estimate technical variability, with the goal of identifying genes that have overall variability that greatly exceeds that expected from technical sources [48, 54, 63]. These methods use spike-ins to estimate technical noise because spike-ins are exposed to most of the same experimental steps as endogenous genes but are free of biological variation. Specifically, Brennecke et al. [48] demonstrated a strong non-linear relationship between gene expression and CV^2^ for spiked-in genes, where CV^2^ represents the square of the coefficient of variation. By modeling this relationship, estimates of technical variability are obtained and genes whose expression variability greatly exceeds these estimates for a given biological variability threshold can be identified. Although useful, this approach does not fully capture cell-to-cell differences in technical variability [63] or give explicit estimates of biological variability [9]. More recent methods provide improvements by estimating biological variability [9] or by incorporating additional aspects of technical noise to estimate parameters that account for variation across cells using spike-ins [63] or jointly over spike-ins and genes [54].

A second group of methods aims to identify and adjust for nuisance variation imposed by oscillatory genes. Specifically, Buettner et al. [47] propose a single-cell latent variable model (scLVM) to adjust for the effects of cell-cycle oscillations. By adjusting for a structured source of variation (resulting from oscillations), the overall residual variance is reduced, increasing the signal-to-noise ratio and effectively increasing power. The scLVM approach estimates a covariance matrix for known cell-cycle genes using a Gaussian-process latent variable model. A linear mixed model is then fitted to each gene with random effects, modeling contributions from hidden factors represented by the covariance matrix, technical noise, and biological variation. Residuals from the fit produce so-called 'corrected' gene expression values in which the variation associated with the cell-cycle has been removed. Buettner et al. [47] demonstrated nicely that previously masked sub-populations associated with T-cell differentiation are revealed following removal of cell cycle-associated variation.

A related approach called Oscope [11] does not rely on oscillating genes being identified a priori. Rather, it was developed to identify and characterize oscillators in snapshot (non temporal) scRNA-seq experiments. When oscillations that are due to the cell cycle or other sources are not of interest but rather are nuisance variables masking the effects that are of interest, the oscillatory gene groups identified by Oscope may be used subsequently in a de-noising step, using either scLVM or, for specific groups of genes, OEFinder [12]. It should be noted that Oscope is useful not only when oscillators are nuisance variables but also when they are of direct interest. For example, Oscope could be used in studies that aim to identify new oscillators (see the “Pseudotemporal ordering and inference” section).

## Sub-population identification

Two of the most common goals of an scRNA-seq experiment are identifying cell sub-populations within a biological condition and characterizing genes that have differential distributions (DD) across conditions. We discuss each separately. As with normalization, the majority of reported scRNA-seq studies use methods developed for bulk experiments. Specifically, hierarchical clustering and/or PCA is often performed on DE [3, 15, 65], highly expressed [66, 67], or highly variable genes [61, 62] (or gene sets [68]) to identify cell sub-populations. A nonlinear dimension-reduction method, t-SNE [69], has also been used for scRNA-seq data and is often applied to a subset of highly variable genes [60–62]. High variability in expression levels among cells of the same type, which is common in scRNA-seq, can cause underlying structure to be undetected by these otherwise useful approaches. In addition, PCA (and related methods such as factor analysis (FA)) can provide misleading results in the single-cell setting because of the presence of zeros [70]. Methods have been developed recently to address these limitations. ZIFA is a dimension-reduction approach that augments a latent variable factor analysis model to accommodate zeros [70]. Pierson and Yau [70] used simulation studies to show that ZIFA has comparable performance to PCA/FA when no (or few) zeros are present and has considerable advantages in the presence of zeros. SNN-Cliq is a computationally efficient clustering approach that relies on shared nearest neighbor (SNN) similarity measures, which utilize rankings of similarities (such as Euclidean distance) between gene expression values as opposed to their numerical values [71]. As the ranking of nodes usually retains meaning in the high-dimensional setting, even when primary similarity measures might not, SNN-cliq proves to be more robust and precise than traditional approaches. Like SNN-cliq, RaceID also provides advantages gained by clustering of processed data as opposed to gene-expression values [59]. Specifically, RaceID performs k-means clustering applied to a similarity matrix determined by Pearson’s correlation coefficients from pairs of cells, which is shown to yield improvements in cluster separation relative to using expression values directly. A second step allows for outlier cells to be regrouped into separate clusters in an effort to identify rare sub-populations. Once sub-populations are identified, it will be very interesting to determine if they correspond to a known cell type; toward this end, a cell-type-enrichment analysis approach similar to gene-set-enrichment analysis has been developed as part of SINCERA [72]. Finally, if data from multiple time points are available, single-cell clustering using bifurcation analysis (SCUBA) can be used to identify sub-populations at an initial time point and to extract lineage relationships between the sub- populations and cells at subsequent time points [73].

## Identifying genes that have expression differences across conditions

To identify genes that have expression differences across conditions in an scRNA-seq study, investigators often use methods from bulk RNA-seq that test for shifts in unimodal distributions across conditions [3, 31, 56, 67, 74, 75]. A number of groups have recognized the deficiencies in doing so: due to both biological and technical variability cell-to-cell, there is often an abundance of cells for which a given gene’s expression is measured at zero. Recent methods, including MAST [76] and SCDE [77], have been developed to accommodate bimodality in expression levels resulting from an abundance of zero (or low) values. In these mixture-model-based approaches, one component distribution accommodates unobserved, or dropout, measurements (which include zero and, optionally, thresholded low-magnitude observations) and a second unimodal component describes gene expression in cells where expression is observed. (Note that SCDE uses a three-component mixture to fit error models and to improve expression estimates but a two-component mixture for testing for differences in expression across conditions.) Although these approaches provide an advance over the unimodal models that are used in the bulk setting, they are insufficient for characterizing multi-modal expression data, which is common in scRNA-seq experiments. For example, cell heterogeneity often gives rise to bimodal distributions within the “observed” component [15, 65].

A recent method developed by Korthauer et al. [78] accommodates multi-modality to identify genes with DD across conditions. In their Bayesian modeling framework, called scDD, they accommodate four types of changes across two biological conditions: shifts in unimodal distributions (traditional DE); differences in the number of modes (DM); differences in the proportion of cells within modes (DP); or both DE and DM, which the authors refer to as DB. The scDD model provides posterior probabilities of DD for each gene and then, using those posterior probabilities, classifies each DD gene into one of the four patterns. By explicitly modeling the multi-modal expression, scDD is likely to have increased power to identify differences in many settings.

## Pseudotemporal ordering and inference

Dynamic processes such as stem cell renewal and differentiation are essential for normal tissue development, homeostasis, and repair, yet our understanding of these fundamental processes remains primitive. Bulk RNA-seq studies have enabled numerous insights, but averaging over thousands of cells obscures, and in some cases misrepresents, signals of interest [79]. Consequently, the ability to profile genome-wide expression in individual cells is critical to improving our understanding of the dynamic cellular processes associated with development, differentiation, and disease. Single-cell RNA-seq experiments provide for such profiling but they too are limited to snapshot experiments, meaning that continuous monitoring of genome-wide gene expression in individual cells over time is not possible. Fortunately, computational algorithms coupled with scRNA-seq data enable reconstruction of differentiation paths from a population of individual unsynchronized cells. The idea is that, at any given time point, an unsynchronized cell population contains cells at various stages of differentiation. (We refer to differentiation throughout, noting that other dynamic biological processes may also be studied using these approaches.) Expression dynamics may be resolved by reordering the cells according to their position along a differentiation path. A number of statistical and computational methods have been developed toward this end.

Although the details differ considerably among methods, most approaches perform some type of dimension reduction and then apply algorithms from graph theory (or extensions thereof) designed to traverse nodes in a graph efficiently. In short, of interest in many graph theoretic considerations is identifying the paths (orderings of nodes) that pass through selected nodes in a graph while minimizing some distance function. This problem is structurally similar to ordering cells along a differentiation path or paths; the goal is to order cells so that the distance between cells, determined by gene expression, is minimized. Typically, distance is defined using genes that explain a substantial proportion of variance or those known to be important in differentiation.

Monocle was the first robust and efficient computational method developed to order cells according to their position along a differentiation process [36]. To reconstruct the so-called pseudotemporal ordering, Monocle uses independent component analysis (ICA) for dimension reduction, then constructs a minimum-spanning tree (MST) through the dimension-reduced data. The longest path through the MST is used initially to place cells according to their progress through differentiation. Divergence among paths is accommodated and numerous trajectories are considered to order cells that are not well placed initially. Monocle’s motivating examples and analysis are done using data from four time points and the authors [36] note that selecting genes that are DE between time points allowed for robust ordering. It is possible to use Monocle for pseudotime ordering of data from a single time point, but doing so would require a user to identify salient, temporally related genes, which in some cases is not possible. Waterfall is similar to Monocle but uses unsupervised clustering and PCA in a pre-processing step to identify groups of ordered cells (temporal delineators are not required) that are then used to reconstruct a full ordering using an MST [80].

Methods that are based on diffusion maps have also been developed [81]. They allow for dimension reduction and reconstruction in a single step, are relatively robust to noise, accommodate zeros, and are largely insensitive to the underlying sampling distribution; but with these advantages comes a considerable computational burden. Specific implementations that consider subsets of paths can dramatically improve performance [82]; and Sincell provides a general implementation in which various options for dimension reduction and pseudotemporal reconstruction may be specified by a user [83].

A key assumption that enables pseudotemporal ordering is that genes do not change direction very often, and thus samples with similar transcriptional profiles should be close in order. If oscillatory processes are of interest, the aforementioned approaches are not appropriate because genes that follow the same oscillatory process need not have similar transcriptional profiles. Two genes with an identical frequency that are phase shifted, for example, will have little similarity. Oscope was developed to enable the identification and reconstruction of oscillatory trajectories [11]. Like other pseudotemporal reconstruction algorithms, Oscope capitalizes on the fact that cells from an unsynchronized population represent distinct states in a system. Unlike previous approaches, however, it does not attempt to construct a linear order that is based on minimizing change among adjacent samples. Instead, it utilizes co-regulation information among oscillators to identify groups of putative oscillating genes and then reconstructs the cyclic order of samples for each group, defined as the order that specifies each cell’s position within one cycle of the oscillation. There are likely other processes of interest whose dynamics are not well described by the pseudotemporal or cyclic order reconstruction methods that are currently available and novel methods in this domain will prove useful.

## Network inference

Elucidating the structure and function of transcriptional regulatory networks is a central goal of numerous studies and scRNA-seq provides unprecedented potential toward this end. This challenge is commonly viewed as a regulatory network reconstruction problem, in which genes or transcripts represent nodes and edges represent interactions or dependence among nodes. Of primary interest are identifying meaningful groups of nodes, estimating edges, and determining the ways in which the network changes following perturbation. The weighted gene co-expression network analysis (WGCNA) is an analysis approach widely used in bulk RNA-seq [84]. In this framework, edges represent co-expression, as determined by both correlation and relative interconnectedness. It is not clear how these measures will perform in general when applied to scRNA-seq, where zeros and sub-populations are common, and it is likely that some adjustment to the existing framework may prove beneficial. At the same time, WGCNA has already enabled important insights in an scRNA-seq study focused on embryonic development [53]. There, the authors applied WGCNA to cells at the same developmental stage, minimizing the potential for spurious results induced by high-correlation coefficients resulting from the presence of sub-populations.

WGCNA and other association network reconstruction approaches have facilitated considerable progress in bulk studies and are expected to do so for scRNA-seq as well, but they do not provide information about regulatory relationships among nodes. To do so, temporal or perturbation experiments are typically required. As described in the previous section, it is possible to derive at least partial ordering from snapshot scRNA-seq experiments; two recent methods leverage the information provided by pseudotemporal ordering approaches and combine it with traditional methods for regulatory network reconstruction to infer regulatory relationships among genes [85, 86]. In doing so, these exciting methods greatly expand upon the type of information that can now be obtained from snapshot scRNA-seq experiments.

## Conclusions

The past decade of genome research has led to major advances in single-cell sequencing technologies and concomitant advances in computational and statistical methodologies. Yet a number of challenges remain and must be addressed to ensure that maximal information can be obtained from single-cell RNA-seq, as well as other types of single-cell experiments. Methods from bulk experiments are readily applicable, perhaps following straightforward extensions, for tasks such as quality control and expression estimation that involve raw data. In contrast, novel methods are required for tasks that are directly affected by features characteristic of single-cell data, including an abundance of zeros, increased heterogeneity, and complex expression distributions. These tasks include normalization, sub-population identification, assessment of differential dynamics, pseudotime reconstruction, and network inference. For questions that involve processed measurements (involving PCA, FA, or network inference methods, for example), a main challenge is how best to define distance. Standard measures of distance may be compromised in the single-cell setting given the frequency of strongly expressed genes that dominate such measures and given the abundance of missing data that are not missing at random but rather arise from both biological and technical sources. Once robust and informative measures of distance are developed, applications of traditional methods will result in much-improved performance.

We have focused primarily on analyses for which multiple tools are available, although we note that there are additional methodological challenges that are not highlighted here. For example, novel methods are beginning to be developed for studies of allele-specific expression [63] and isoform usage [87]. Methodological challenges are also introduced with the advent of technologies that increase sample size by allowing for routine profiling of tens of thousands of cells [61, 62, 88]. Some of the methods discussed here will enjoy improved performance, whereas others will require extensions or approximations to handle the computational burden. Advances will also facilitate the study of single-cell gene-expression profiles over time, space [89], or lineage [90], as well as of multiple types of -omics data within a single cell [91]. This progress will usher in unique opportunities to fully address fundamental questions associated with cell state, circuitry, and fate decisions during development, homeostasis, and disease. As we enter a period of unparalleled data accumulation and analysis, computational biology will undoubtedly continue to contribute important advances to our understanding of molecular systems.

## Data

Six publically available datasets (three bulk and three single cell) are shown in Fig. 1. Datasets bulk1, bulk3, and sc3 contained spike-ins that were removed prior to analysis. The six datasets are described in detail below.

### Bulk1

Gene-level read counts calculated by HTSeq were downloaded from GEO GSE60314, genome release 6.01. In short, RNA was extracted from individual *Drosophila* Genetic Reference Panel (DGRP) flies and then multiplexed and sequenced using the Illumina HiSeq 2000 system. Here, we consider 60 female bulk RNA-seq samples to match the number of samples in the sc1 set. To make the sequencing depths comparable, for each sc1 cell, a sample having comparable sequencing depth was drawn from the 851 available bulk1 samples. The 60 chosen bulk samples have an average sequencing depth of nine million and were normalized using the median normalization method [42].

### Bulk2

Transcript-level RPKM expression estimates were downloaded from GEO GSE40419. In short, RNA was extracted from tissue containing normal lung cells; cDNA was prepared using the standard Illumina protocol and sequenced using the Illumina HiSeq 2000 system. The data contain 77 bulk RNA-seq samples. Prior to RPKM normalization, reads were aligned using GSNAP and gene-level counts were quantified by counting the number of uniquely aligned RNA sequencing reads.

### Bulk3

Transcript-level read counts were downloaded from GEO GSE47774. Here we consider 80 replicate samples of Universal Human Reference (UHR) RNA (sample A) sequenced at Beijing Genomics Institute (BGI) as part of the Sequencing Quality Control (SEQC) project. Five barcoded replicate libraries were generated then multiplexed and sequenced across eight lanes for two flow cells using the Illumina HiSeq 2000 system. The data have an average sequencing depth of 13.4 million and were normalized using the median normalization method [42].

### Sc1

Gene-level read counts (generated using rpkmforgenes) were downloaded from GEO GSE45719. RNA was collected from individual *Mus musculus* embryonic cells at various time points of development. Cells were handpicked and libraries were prepared using the Smart-seq protocol. Libraries were multiplexed and sequenced on the Illumina HiSeq 2000 system. Here we consider scRNA-seq of 60 cells from the mid blastocyst embryo stage. The data have an average sequencing depth of nine million and were normalized using the median normalization method [42].

### Sc2

TPM expression estimates generated by RSEM were downloaded from GEO GSE64016. In short, RNA was extracted from undifferentiated H1 human embryonic stem cells in three replicate experiments. Single-cell RNA-seq was performed using the C1 Single Cell Auto Prep System (Fluidigm); libraries were then multiplexed and sequenced at 24 libraries per lane on the Illumina HiSeq 2500 system. The data have an average sequencing depth of 3.4 million.

### Sc3

Gene-level read counts calculated by HTSeq were downloaded from ArrayExpress E-MTAB- 2805. In short, individual *Mus musculus* embryonic stem cells were sorted using fluorescence-activated cell sorting (FACS) for cell-cycle stage, then single cell RNA-seq was performed using the C1 Single Cell Auto Prep System (Fluidigm). Libraries were multiplexed and sequenced across four lanes using the Illumina HiSeq 2000 system. Here we consider 96 *Mus musculus* embryonic stem cells in the G_2_M stage of the cell cycle. The data have an average sequencing depth of 4.5 million and were normalized using the median normalization method [42].

## Abbreviations

CV^2^: the square of the coefficient of variation
DD: differential distribution
DE: differentially expressed
DM: differences in the number of modes
DP: differences in the proportion of cells within modes
FA: factor analysis
FPKM: fragments per kilobase per million mapped reads
ICA: independent component analysis
MST: minimum-spanning tree
PCA: principal component analysis
RPKM: reads per kilobase per million mapped reads
scLVM: single-cell latent variable model
scRNA-seq: single-cell RNA-sequencing
SCUBA: single-cell clustering using bifurcation analysis
SNN: shared nearest neighbor
TPM: transcripts per million mapped reads
UMI: unique molecular identifier
WGCNA: weighted gene co-expression network analysis
